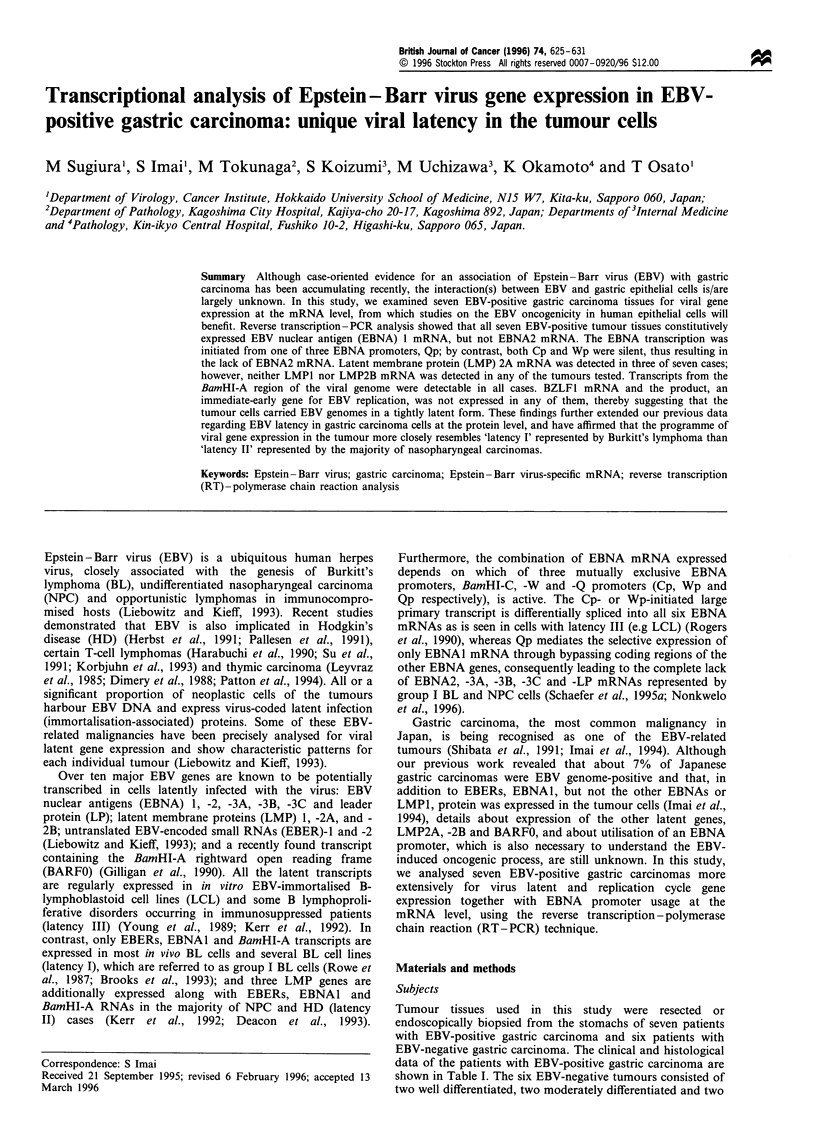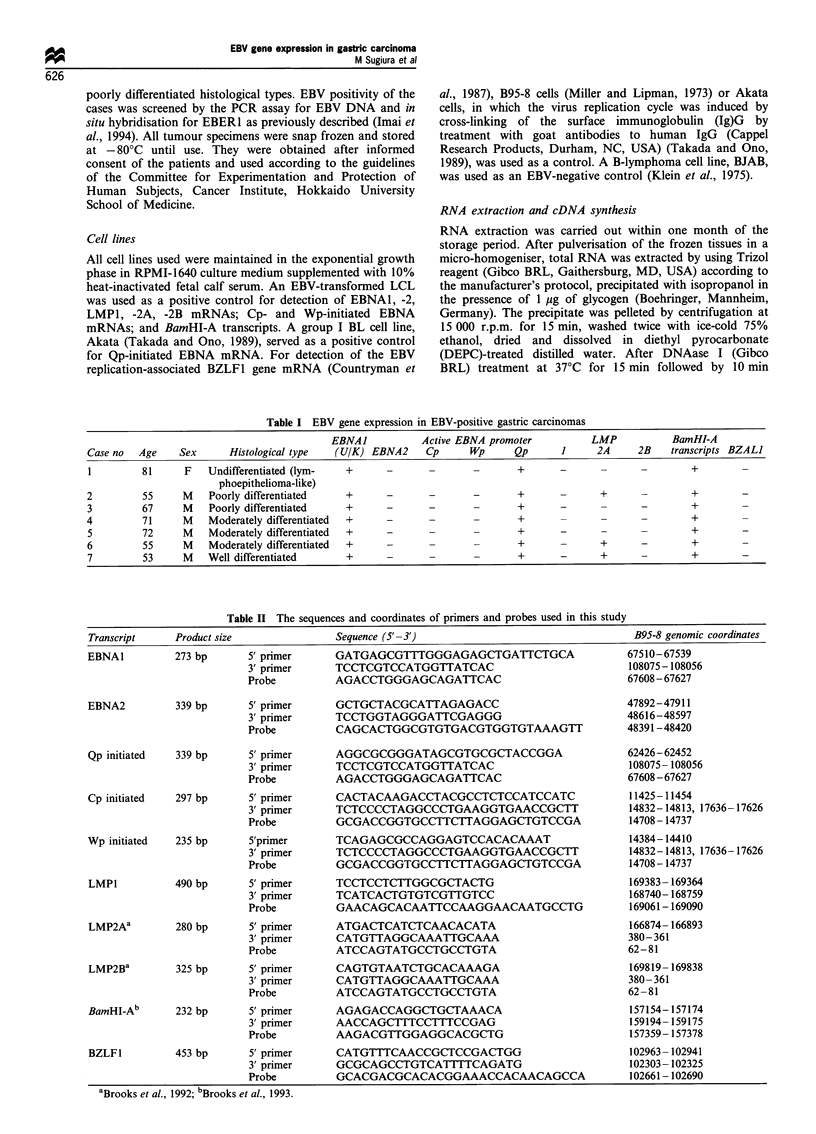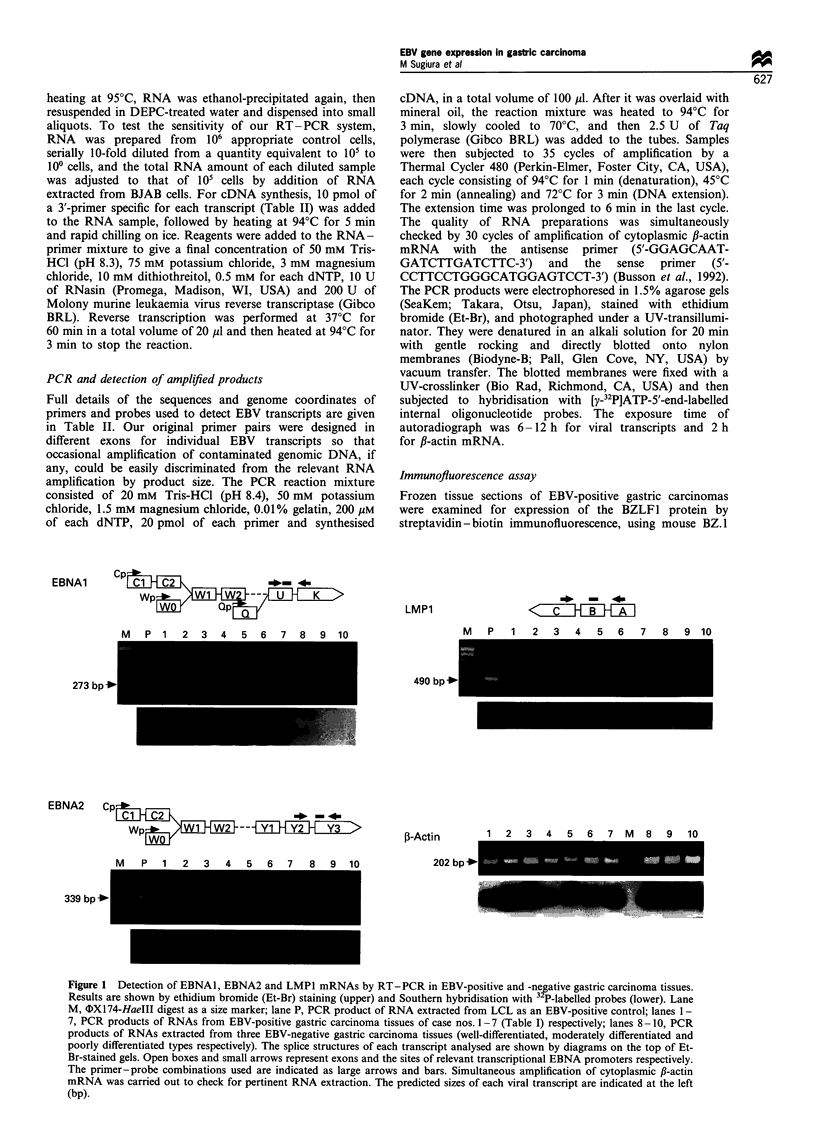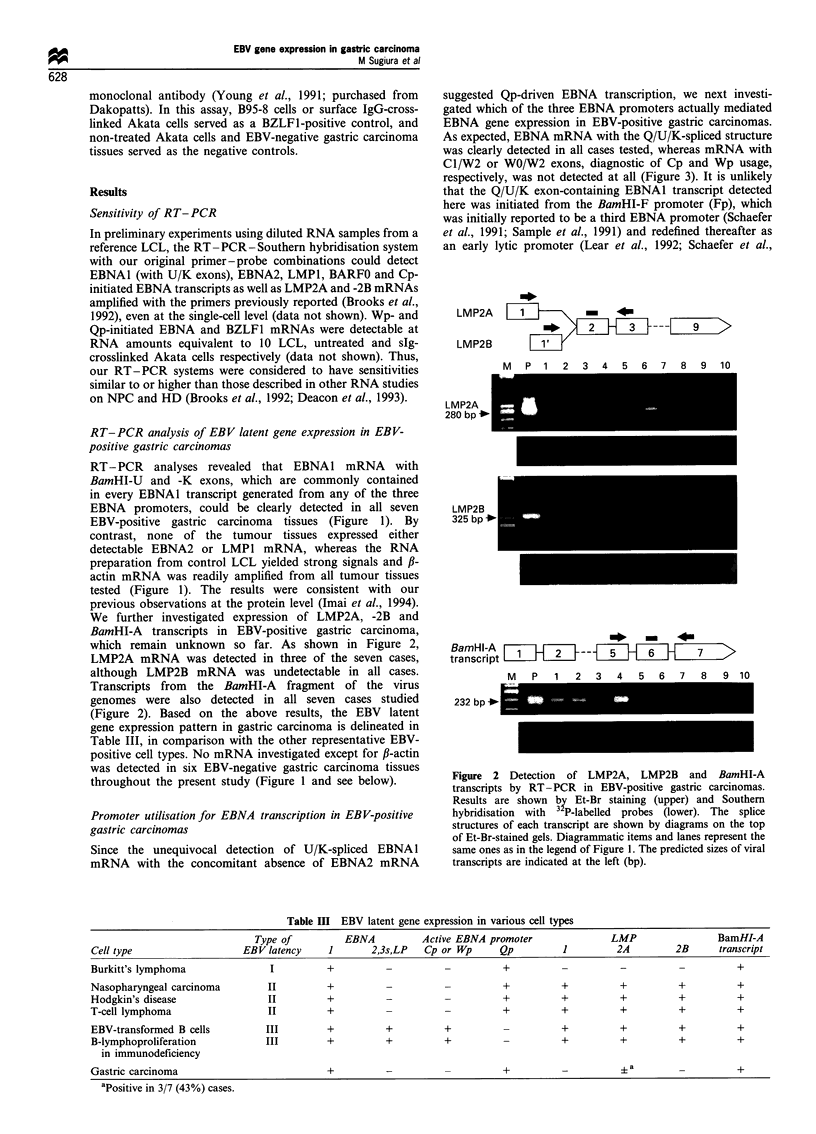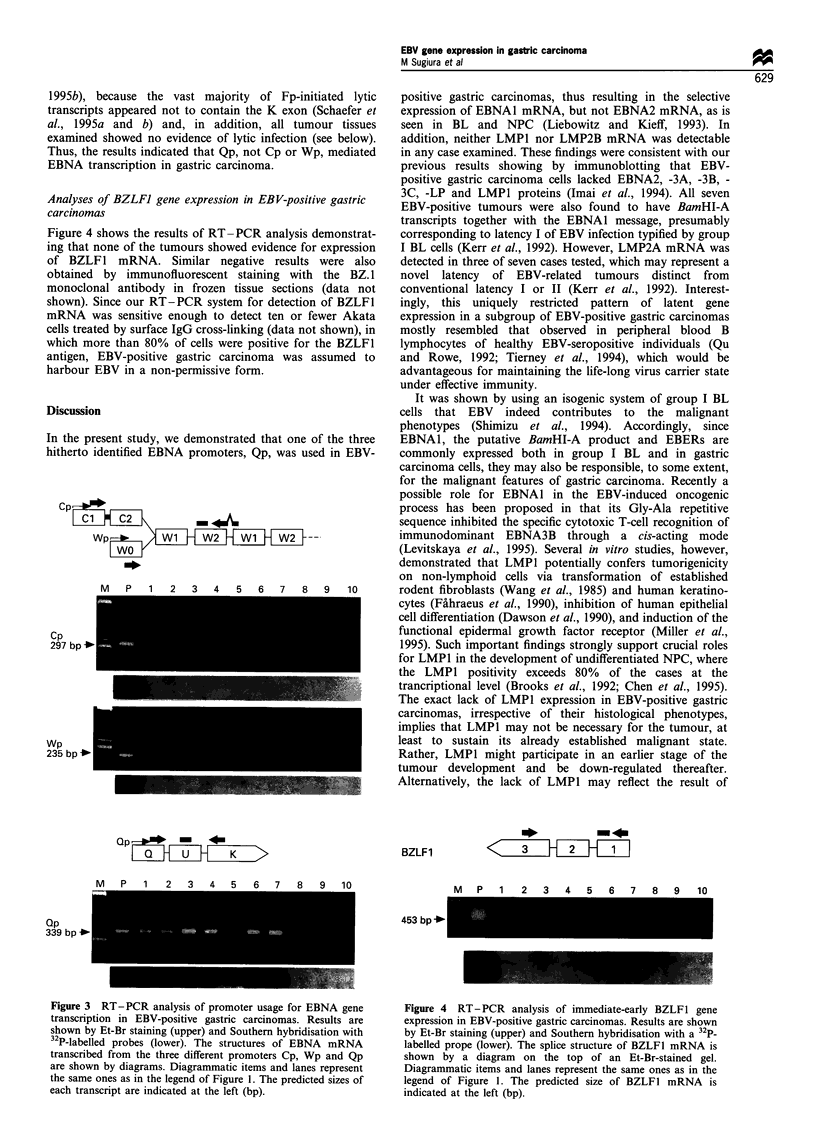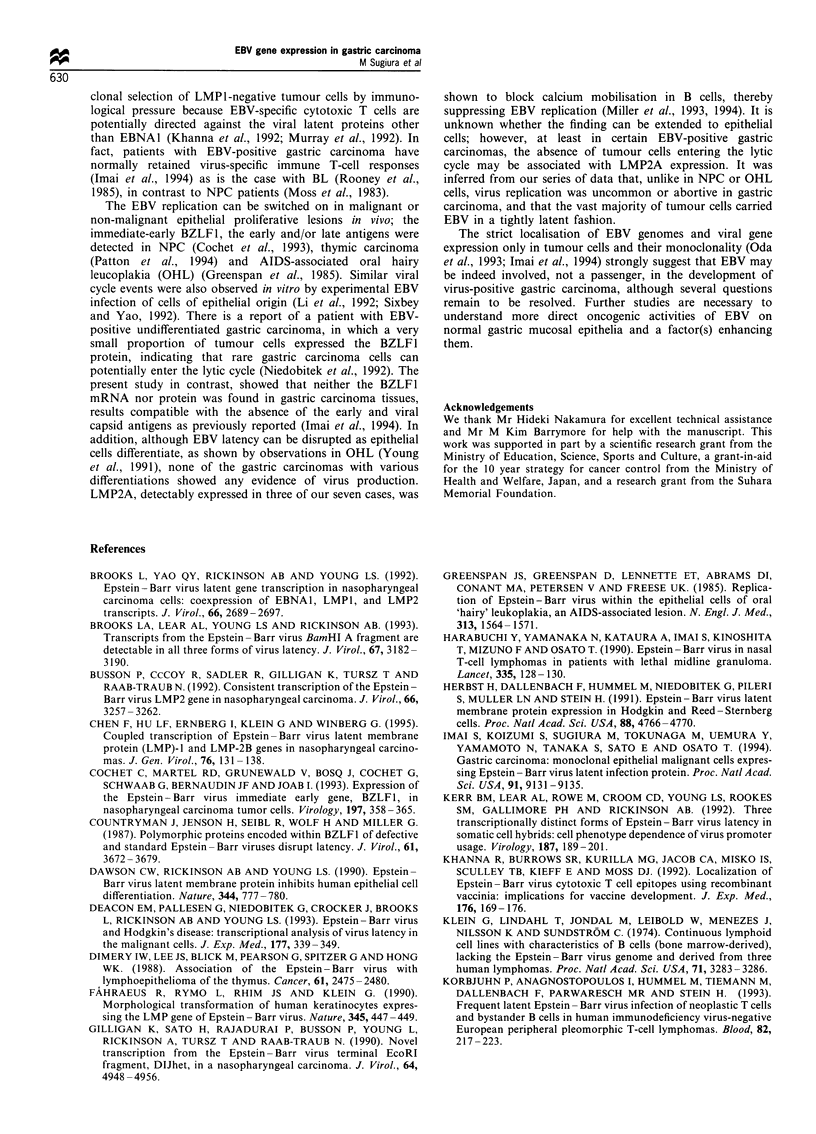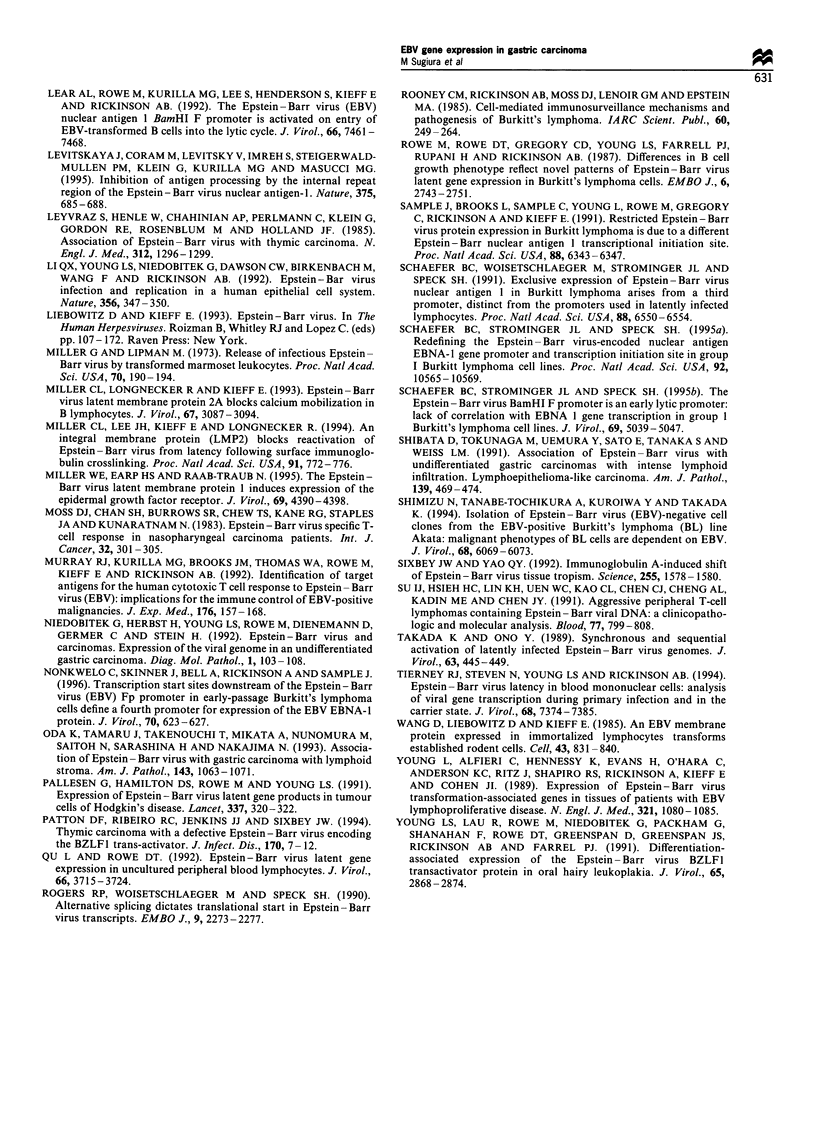# Transcriptional analysis of Epstein-Barr virus gene expression in EBV-positive gastric carcinoma: unique viral latency in the tumour cells.

**DOI:** 10.1038/bjc.1996.412

**Published:** 1996-08

**Authors:** M. Sugiura, S. Imai, M. Tokunaga, S. Koizumi, M. Uchizawa, K. Okamoto, T. Osato

**Affiliations:** Department of Virology, Hokkaido University School of Medicine, Sapporo, Japan.

## Abstract

**Images:**


British Journal of Cancer (1996) 74, 625-631
© 1996 Stockton Press All rights reserved 0007-0920/96 $12.00

### Transcriptional analysis of Epstein-Barr virus gene expression in EBV-positive gastric carcinoma: unique viral latency in the tumour cells

M Sugiura[1], S Imai[1], M Tokunaga[2], S Koizumi[3], M Uchizawa[3], K Okamoto[4] and T Osato[1]

[1]*Department of Virology, Cancer Institute, Hokkaido University School of Medicine, N15 W7, Kita-ku, Sapporo 060, Japan;* [2]*Department of Pathology, Kagoshima City Hospital, Kajiya-cho 20-17, Kagoshima 892, Japan; Departments of* [3]*Internal Medicine and* [4]*Pathology, Kin-ikyo Central Hospital, Fushiko 10-2, Higashi-ku, Sapporo 065, Japan.*

**Summary** Although case-oriented evidence for an association of Epstein-Barr virus (EBV) with gastric carcinoma has been accumulating recently, the interaction(s) between EBV and gastric epithelial cells is/are largely unknown. In this study, we examined seven EBV-positive gastric carcinoma tissues for viral gene expression at the mRNA level, from which studies on the EBV oncogenicity in human epithelial cells will benefit. Reverse transcription-PCR analysis showed that all seven EBV-positive tumour tissues constitutively expressed EBV nuclear antigen (EBNA) 1 mRNA, but not EBNA2 mRNA. The EBNA transcription was initiated from one of three EBNA promoters, Qp; by contrast, both Cp and Wp were silent, thus resulting in the lack of EBNA2 mRNA. Latent membrane protein (LMP) 2A mRNA was detected in three of seven cases; however, neither LMP1 nor LMP2B mRNA was detected in any of the tumours tested. Transcripts from the *Bam*HI-A region of the viral genome were detectable in all cases. BZLF1 mRNA and the product, an immediate-early gene for EBV replication, was not expressed in any of them, thereby suggesting that the tumour cells carried EBV genomes in a tightly latent form. These findings further extended our previous data regarding EBV latency in gastric carcinoma cells at the protein level, and have affirmed that the programme of viral gene expression in the tumour more closely resembles 'latency I' represented by Burkitt's lymphoma than 'latency II' represented by the majority of nasopharyngeal carcinomas.

**Keywords:** Epstein-Barr virus; gastric carcinoma; Epstein-Barr virus-specific mRNA; reverse transcription (RT)-polymerase chain reaction analysis

Epstein-Barr virus (EBV) is a ubiquitous human herpes virus, closely associated with the genesis of Burkitt's lymphoma (BL), undifferentiated nasopharyngeal carcinoma (NPC) and opportunistic lymphomas in immunocompromised hosts (Liebowitz and Kieff, 1993). Recent studies demonstrated that EBV is also implicated in Hodgkin's disease (HD) (Herbst *et al.*, 1991; Pallesen *et al.*, 1991), certain T-cell lymphomas (Harabuchi *et al.*, 1990; Su *et al.*, 1991; Korbjuhn *et al.*, 1993) and thymic carcinoma (Leyvraz *et al.*, 1985; Dimery *et al.*, 1988; Patton *et al.*, 1994). All or a significant proportion of neoplastic cells of the tumours harbour EBV DNA and express virus-coded latent infection (immortalisation-associated) proteins. Some of these EBV-related malignancies have been precisely analysed for viral latent gene expression and show characteristic patterns for each individual tumour (Liebowitz and Kieff, 1993).

Over ten major EBV genes are known to be potentially transcribed in cells latently infected with the virus: EBV nuclear antigens (EBNA) 1, -2, -3A, -3B, -3C and leader protein (LP); latent membrane proteins (LMP) 1, -2A, and -2B; untranslated EBV-encoded small RNAs (EBER)-1 and -2 (Liebowitz and Kieff, 1993); and a recently found transcript containing the *Bam*HI-A rightward open reading frame (BARF0) (Gilligan *et al.*, 1990). All the latent transcripts are regularly expressed in *in vitro* EBV-immortalised B-lymphoblastoid cell lines (LCL) and some B lymphoproliferative disorders occurring in immunosuppressed patients (latency III) (Young *et al.*, 1989; Kerr *et al.*, 1992). In contrast, only EBERs, EBNA1 and *Bam*HI-A transcripts are expressed in most *in vivo* BL cells and several BL cell lines (latency I), which are referred to as group I BL cells (Rowe *et al.*, 1987; Brooks *et al.*, 1993); and three LMP genes are additionally expressed along with EBERs, EBNA1 and *Bam*HI-A RNAs in the majority of NPC and HD (latency II) cases (Kerr *et al.*, 1992; Deacon *et al.*, 1993). Furthermore, the combination of EBNA mRNA expressed depends on which of three mutually exclusive EBNA promoters, *Bam*HI-C, -W and -Q promoters (Cp, Wp and Qp respectively), is active. The Cp- or Wp-initiated large primary transcript is differentially spliced into all six EBNA mRNAs as is seen in cells with latency III (e.g LCL) (Rogers *et al.*, 1990), whereas Qp mediates the selective expression of only EBNA1 mRNA through bypassing coding regions of the other EBNA genes, consequently leading to the complete lack of EBNA2, -3A, -3B, -3C and -LP mRNAs represented by group I BL and NPC cells (Schaefer *et al.*, 1995*a*; Nonkwelo *et al.*, 1996).

Gastric carcinoma, the most common malignancy in Japan, is being recognised as one of the EBV-related tumours (Shibata *et al.*, 1991; Imai *et al.*, 1994). Although our previous work revealed that about 7% of Japanese gastric carcinomas were EBV genome-positive and that, in addition to EBERs, EBNA1, but not the other EBNAs or LMP1, protein was expressed in the tumour cells (Imai *et al.*, 1994), details about expression of the other latent genes, LMP2A, -2B and BARF0, and about utilisation of an EBNA promoter, which is also necessary to understand the EBV-induced oncogenic process, are still unknown. In this study, we analysed seven EBV-positive gastric carcinomas more extensively for virus latent and replication cycle gene expression together with EBNA promoter usage at the mRNA level, using the reverse transcription-polymerase chain reaction (RT-PCR) technique.

### Materials and methods

### *Subjects*

Tumour tissues used in this study were resected or endoscopically biopsied from the stomachs of seven patients with EBV-positive gastric carcinoma and six patients with EBV-negative gastric carcinoma. The clinical and histological data of the patients with EBV-positive gastric carcinoma are shown in Table I. The six EBV-negative tumours consisted of two well differentiated, two moderately differentiated and two

Correspondence: S Imai
Received 21 September 1995; revised 6 February 1996; accepted 13 March 1996

poorly differentiated histological types. EBV positivity of the cases was screened by the PCR assay for EBV DNA and *in situ* hybridisation for EBER1 as previously described (Imai *et al.*, 1994). All tumour specimens were snap frozen and stored at −80°C until use. They were obtained after informed consent of the patients and used according to the guidelines of the Committee for Experimentation and Protection of Human Subjects, Cancer Institute, Hokkaido University School of Medicine.

### *Cell lines*

All cell lines used were maintained in the exponential growth phase in RPMI-1640 culture medium supplemented with 10% heat-inactivated fetal calf serum. An EBV-transformed LCL was used as a positive control for detection of EBNA1, -2, LMP1, -2A, -2B mRNAs; Cp- and Wp-initiated EBNA mRNAs; and *Bam*HI-A transcripts. A group I BL cell line, Akata (Takada and Ono, 1989), served as a positive control for Qp-initiated EBNA mRNA. For detection of the EBV replication-associated BZLF1 gene mRNA (Countryman *et al.*, 1987), B95-8 cells (Miller and Lipman, 1973) or Akata cells, in which the virus replication cycle was induced by cross-linking of the surface immunoglobulin (Ig)G by treatment with goat antibodies to human IgG (Cappel Research Products, Durham, NC, USA) (Takada and Ono, 1989), was used as a control. A B-lymphoma cell line, BJAB, was used as an EBV-negative control (Klein *et al.*, 1975).

### *RNA extraction and cDNA synthesis*

RNA extraction was carried out within one month of the storage period. After pulverisation of the frozen tissues in a micro-homogeniser, total RNA was extracted by using Trizol reagent (Gibco BRL, Gaithersburg, MD, USA) according to the manufacturer's protocol, precipitated with isopropanol in the pressence of 1 μg of glycogen (Boehringer, Mannheim, Germany). The precipitate was pelleted by centrifugation at 15 000 r.p.m. for 15 min, washed twice with ice-cold 75% ethanol, dried and dissolved in diethyl pyrocarbonate (DEPC)-treated distilled water. After DNAase I (Gibco BRL) treatment at 37°C for 15 min followed by 10 min

**Table I** EBV gene expression in EBV-positive gastric carcinomas

| Case no | Age | Sex | Histological type | EBNA1 (U/K) | EBNA2 | Active EBNA promoter Cp | Wp | Qp | LMP 1 | 2A | 2B | BamHI-A transcripts | BZAL1 |
|---|---|---|---|---|---|---|---|---|---|---|---|---|---|
| 1 | 81 | F | Undifferentiated (lymphoepithelioma-like) | + | − | − | − | + | − | − | − | + | − |
| 2 | 55 | M | Poorly differentiated | + | − | − | − | + | − | + | − | + | − |
| 3 | 67 | M | Poorly differentiated | + | − | − | − | + | − | − | − | + | − |
| 4 | 71 | M | Moderately differentiated | + | − | − | − | + | − | − | − | + | − |
| 5 | 72 | M | Moderately differentiated | + | − | − | − | + | − | − | − | + | − |
| 6 | 55 | M | Moderately differentiated | + | − | − | − | + | − | + | − | + | − |
| 7 | 53 | M | Well differentiated | + | − | − | − | + | − | + | − | + | − |

**Table II** The sequences and coordinates of primers and probes used in this study

| Transcript | Product size | | Sequence (5′−3′) | B95-8 genomic coordinates |
|---|---|---|---|---|
| EBNA1 | 273 bp | 5′ primer | GATGAGCGTTTGGGAGAGCTGATTCTGCA | 67510−67539 |
| | | 3′ primer | TCCTCGTCCATGGTTATCAC | 108075−108056 |
| | | Probe | AGACCTGGGAGCAGATTCAC | 67608−67627 |
| EBNA2 | 339 bp | 5′ primer | GCTGCTACGCATTAGAGACC | 47892−47911 |
| | | 3′ primer | TCCTGGTAGGGATTCGAGGG | 48616−48597 |
| | | Probe | CAGCACTGGCGTGTGACGTGGTGTAAAGTT | 48391−48420 |
| Qp initiated | 339 bp | 5′ primer | AGGCGCGGGATAGCGTGCGCTACCGGA | 62426−62452 |
| | | 3′ primer | TCCTCGTCCATGGTTATCAC | 108075−108056 |
| | | Probe | AGACCTGGGAGCAGATTCAC | 67608−67627 |
| Cp initiated | 297 bp | 5′ primer | CACTACAAGACCTACGCCTCTCCATCCATC | 11425−11454 |
| | | 3′ primer | TCTCCCCTAGGCCCTGAAGGTGAACCGCTT | 14832−14813, 17636−17626 |
| | | Probe | GCGACCGGTGCCTTCTTAGGAGCTGTCCGA | 14708−14737 |
| Wp initiated | 235 bp | 5′primer | TCAGAGCGCCAGGAGTCCACACAAAT | 14384−14410 |
| | | 3′ primer | TCTCCCCTAGGCCCTGAAGGTGAACCGCTT | 14832−14813, 17636−17626 |
| | | Probe | GCGACCGGTGCCTTCTTAGGAGCTGTCCGA | 14708−14737 |
| LMP1 | 490 bp | 5′ primer | TCCTCCTCTTGGCGCTACTG | 169383−169364 |
| | | 3′ primer | TCATCACTGTGTCGTTGTCC | 168740−168759 |
| | | Probe | GAACAGCACAATTCCAAGGAACAATGCCTG | 169061−169090 |
| LMP2A[a] | 280 bp | 5′ primer | ATGACTCATCTCAACACATA | 166874−166893 |
| | | 3′ primer | CATGTTAGGCAAATTGCAAA | 380−361 |
| | | Probe | ATCCAGTATGCCTGCCTGTA | 62−81 |
| LMP2B[a] | 325 bp | 5′ primer | CAGTGTAATCTGCACAAAGA | 169819−169838 |
| | | 3′ primer | CATGTTAGGCAAATTGCAAA | 380−361 |
| | | Probe | ATCCAGTATGCCTGCCTGTA | 62−81 |
| *Bam*HI-A[b] | 232 bp | 5′ primer | AGAGACCAGGCTGCTAAACA | 157154−157174 |
| | | 3′ primer | AACCAGCTTTCCTTTCCGAG | 159194−159175 |
| | | Probe | AAGACGTTGGAGGCACGCTG | 157359−157378 |
| BZLF1 | 453 bp | 5′ primer | CATGTTTCAACCGCTCCGACTGG | 102963−102941 |
| | | 3′ primer | GCGCAGCCTGTCATTTTCAGATG | 102303−102325 |
| | | Probe | GCACGACGCACACGGAAACCACAACAGCCA | 102661−102690 |

[a]Brooks *et al.*, 1992; [b]Brooks *et al.*, 1993.

heating at 95°C, RNA was ethanol-precipitated again, then resuspended in DEPC-treated water and dispensed into small aliquots. To test the sensitivity of our RT–PCR system, RNA was prepared from $10^6$ appropriate control cells, serially 10-fold diluted from a quantity equivalent to $10^5$ to $10^0$ cells, and the total RNA amount of each diluted sample was adjusted to that of $10^5$ cells by addition of RNA extracted from BJAB cells. For cDNA synthesis, 10 pmol of a 3′-primer specific for each transcript (Table II) was added to the RNA sample, followed by heating at 94°C for 5 min and rapid chilling on ice. Reagents were added to the RNA–primer mixture to give a final concentration of 50 mM Tris-HCl (pH 8.3), 75 mM potassium chloride, 3 mM magnesium chloride, 10 mM dithiothreitol, 0.5 mM for each dNTP, 10 U of RNasin (Promega, Madison, WI, USA) and 200 U of Molony murine leukaemia virus reverse transcriptase (Gibco BRL). Reverse transcription was performed at 37°C for 60 min in a total volume of 20 µl and then heated at 94°C for 3 min to stop the reaction.

### *PCR and detection of amplified products*

Full details of the sequences and genome coordinates of primers and probes used to detect EBV transcripts are given in Table II. Our original primer pairs were designed in different exons for individual EBV transcripts so that occasional amplification of contaminated genomic DNA, if any, could be easily discriminated from the relevant RNA amplification by product size. The PCR reaction mixture consisted of 20 mM Tris-HCl (pH 8.4), 50 mM potassium chloride, 1.5 mM magnesium chloride, 0.01% gelatin, 200 µM of each dNTP, 20 pmol of each primer and synthesised cDNA, in a total volume of 100 µl. After it was overlaid with mineral oil, the reaction mixture was heated to 94°C for 3 min, slowly cooled to 70°C, and then 2.5 U of *Taq* polymerase (Gibco BRL) was added to the tubes. Samples were then subjected to 35 cycles of amplification by a Thermal Cycler 480 (Perkin-Elmer, Foster City, CA, USA), each cycle consisting of 94°C for 1 min (denaturation), 45°C for 2 min (annealing) and 72°C for 3 min (DNA extension). The extension time was prolonged to 6 min in the last cycle. The quality of RNA preparations was simultaneously checked by 30 cycles of amplification of cytoplasmic β-actin mRNA with the antisense primer (5′-GGAGCAAT-GATCTTGATCTTC-3′) and the sense primer (5′-CCTTCCTGGGCATGGAGTCCT-3′) (Busson *et al.*, 1992). The PCR products were electrophoresed in 1.5% agarose gels (SeaKem; Takara, Otsu, Japan), stained with ethidium bromide (Et-Br), and photographed under a UV-transilluminator. They were denatured in an alkali solution for 20 min with gentle rocking and directly blotted onto nylon membranes (Biodyne-B; Pall, Glen Cove, NY, USA) by vacuum transfer. The blotted membranes were fixed with a UV-crosslinker (Bio Rad, Richmond, CA, USA) and then subjected to hybridisation with [$\gamma$-$^{32}$P]ATP-5′-end-labelled internal oligonucleotide probes. The exposure time of autoradiograph was 6–12 h for viral transcripts and 2 h for β-actin mRNA.

### *Immunofluorescence assay*

Frozen tissue sections of EBV-positive gastric carcinomas were examined for expression of the BZLF1 protein by streptavidin–biotin immunofluorescence, using mouse BZ.1

**Figure 1** Detection of EBNA1, EBNA2 and LMP1 mRNAs by RT–PCR in EBV-positive and -negative gastric carcinoma tissues. Results are shown by ethidium bromide (Et-Br) staining (upper) and Southern hybridisation with $^{32}$P-labelled probes (lower). Lane M, ΦX174-*Hae*III digest as a size marker; lane P, PCR product of RNA extracted from LCL as an EBV-positive control; lanes 1–7, PCR products of RNAs from EBV-positive gastric carcinoma tissues of case nos. 1–7 (Table I) respectively; lanes 8–10, PCR products of RNAs extracted from three EBV-negative gastric carcinoma tissues (well-differentiated, moderately differentiated and poorly differentiated types respectively). The splice structures of each transcript analysed are shown by diagrams on the top of Et-Br-stained gels. Open boxes and small arrows represent exons and the sites of relevant transcriptional EBNA promoters respectively. The primer–probe combinations used are indicated as large arrows and bars. Simultaneous amplification of cytoplasmic β-actin mRNA was carried out to check for pertinent RNA extraction. The predicted sizes of each viral transcript are indicated at the left (bp).

monoclonal antibody (Young *et al.*, 1991; purchased from Dakopatts). In this assay, B95-8 cells or surface IgG-cross-linked Akata cells served as a BZLF1-positive control, and non-treated Akata cells and EBV-negative gastric carcinoma tissues served as the negative controls.

### Results

### *Sensitivity of RT–PCR*

In preliminary experiments using diluted RNA samples from a reference LCL, the RT–PCR–Southern hybridisation system with our original primer–probe combinations could detect EBNA1 (with U/K exons), EBNA2, LMP1, BARF0 and Cp-initiated EBNA transcripts as well as LMP2A and -2B mRNAs amplified with the primers previously reported (Brooks *et al.*, 1992), even at the single-cell level (data not shown). Wp- and Qp-initiated EBNA and BZLF1 mRNAs were detectable at RNA amounts equivalent to 10 LCL, untreated and sIg-crosslinked Akata cells respectively (data not shown). Thus, our RT–PCR systems were considered to have sensitivities similar to or higher than those described in other RNA studies on NPC and HD (Brooks *et al.*, 1992; Deacon *et al.*, 1993).

### *RT–PCR analysis of EBV latent gene expression in EBV-positive gastric carcinomas*

RT–PCR analyses revealed that EBNA1 mRNA with *Bam*HI-U and -K exons, which are commonly contained in every EBNA1 transcript generated from any of the three EBNA promoters, could be clearly detected in all seven EBV-positive gastric carcinoma tissues (Figure 1). By contrast, none of the tumour tissues expressed either detectable EBNA2 or LMP1 mRNA, whereas the RNA preparation from control LCL yielded strong signals and β-actin mRNA was readily amplified from all tumour tissues tested (Figure 1). The results were consistent with our previous observations at the protein level (Imai *et al.*, 1994). We further investigated expression of LMP2A, -2B and *Bam*HI-A transcripts in EBV-positive gastric carcinoma, which remain unknown so far. As shown in Figure 2, LMP2A mRNA was detected in three of the seven cases, although LMP2B mRNA was undetectable in all cases. Transcripts from the *Bam*HI-A fragment of the virus genomes were also detected in all seven cases studied (Figure 2). Based on the above results, the EBV latent gene expression pattern in gastric carcinoma is delineated in Table III, in comparison with the other representative EBV-positive cell types. No mRNA investigated except for β-actin was detected in six EBV-negative gastric carcinoma tissues throughout the present study (Figure 1 and see below).

### *Promoter utilisation for EBNA transcription in EBV-positive gastric carcinomas*

Since the unequivocal detection of U/K-spliced EBNA1 mRNA with the concomitant absence of EBNA2 mRNA suggested Qp-driven EBNA transcription, we next investigated which of the three EBNA promoters actually mediated EBNA gene expression in EBV-positive gastric carcinomas. As expected, EBNA mRNA with the Q/U/K-spliced structure was clearly detected in all cases tested, whereas mRNA with C1/W2 or W0/W2 exons, diagnostic of Cp and Wp usage, respectively, was not detected at all (Figure 3). It is unlikely that the Q/U/K exon-containing EBNA1 transcript detected here was initiated from the *Bam*HI-F promoter (Fp), which was initially reported to be a third EBNA promoter (Schaefer *et al.*, 1991; Sample *et al.*, 1991) and redefined thereafter as an early lytic promoter (Lear *et al.*, 1992; Schaefer *et al.*,

**Figure 2** Detection of LMP2A, LMP2B and *Bam*HI-A transcripts by RT–PCR in EBV-positive gastric carcinomas. Results are shown by Et-Br staining (upper) and Southern hybridisation with $^{32}$P-labelled probes (lower). The splice structures of each transcript are shown by diagrams on the top of Et-Br-stained gels. Diagrammatic items and lanes represent the same ones as in the legend of Figure 1. The predicted sizes of viral transcripts are indicated at the left (bp).

**Table III** EBV latent gene expression in various cell types

| Cell type | Type of EBV latency | EBNA 1 | EBNA 2,3s,LP | Active EBNA promoter Cp or Wp | Active EBNA promoter Qp | LMP 1 | LMP 2A | LMP 2B | BamHI-A transcript |
|---|---|---|---|---|---|---|---|---|---|
| Burkitt's lymphoma | I | + | – | – | + | – | – | – | + |
| Nasopharyngeal carcinoma | II | + | – | – | + | + | + | + | + |
| Hodgkin's disease | II | + | – | – | + | + | + | + | + |
| T-cell lymphoma | II | + | – | – | + | + | + | + | + |
| EBV-transformed B cells | III | + | + | + | – | + | + | + | + |
| B-lymphoproliferation in immunodeficiency | III | + | + | + | – | + | + | + | + |
| Gastric carcinoma | | + | – | – | + | – | ±[a] | – | + |

[a]Positive in 3/7 (43%) cases.

1995*b*), because the vast majority of Fp-initiated lytic transcripts appeared not to contain the K exon (Schaefer *et al.*, 1995*a* and *b*) and, in addition, all tumour tissues examined showed no evidence of lytic infection (see below). Thus, the results indicated that Qp, not Cp or Wp, mediated EBNA transcription in gastric carcinoma.

*Analyses of BZLF1 gene expression in EBV-positive gastric carcinomas*

Figure 4 shows the results of RT–PCR analysis demonstrating that none of the tumours showed evidence for expression of BZLF1 mRNA. Similar negative results were also obtained by immunofluorescent staining with the BZ.1 monoclonal antibody in frozen tissue sections (data not shown). Since our RT–PCR system for detection of BZLF1 mRNA was sensitive enough to detect ten or fewer Akata cells treated by surface IgG cross-linking (data not shown), in which more than 80% of cells were positive for the BZLF1 antigen, EBV-positive gastric carcinoma was assumed to harbour EBV in a non-permissive form.

### Discussion

In the present study, we demonstrated that one of the three hitherto identified EBNA promoters, Qp, was used in EBV-positive gastric carcinomas, thus resulting in the selective expression of EBNA1 mRNA, but not EBNA2 mRNA, as is seen in BL and NPC (Liebowitz and Kieff, 1993). In addition, neither LMP1 nor LMP2B mRNA was detectable in any case examined. These findings were consistent with our previous results showing by immunoblotting that EBV-positive gastric carcinoma cells lacked EBNA2, -3A, -3B, -3C, -LP and LMP1 proteins (Imai *et al.*, 1994). All seven EBV-positive tumours were also found to have *Bam*HI-A transcripts together with the EBNA1 message, presumably corresponding to latency I of EBV infection typified by group I BL cells (Kerr *et al.*, 1992). However, LMP2A mRNA was detected in three of seven cases tested, which may represent a novel latency of EBV-related tumours distinct from conventional latency I or II (Kerr *et al.*, 1992). Interestingly, this uniquely restricted pattern of latent gene expression in a subgroup of EBV-positive gastric carcinomas mostly resembled that observed in peripheral blood B lymphocytes of healthy EBV-seropositive individuals (Qu and Rowe, 1992; Tierney *et al.*, 1994), which would be advantageous for maintaining the life-long virus carrier state under effective immunity.

It was shown by using an isogenic system of group I BL cells that EBV indeed contributes to the malignant phenotypes (Shimizu *et al.*, 1994). Accordingly, since EBNA1, the putative *Bam*HI-A product and EBERs are commonly expressed both in group I BL and in gastric carcinoma cells, they may also be responsible, to some extent, for the malignant features of gastric carcinoma. Recently a possible role for EBNA1 in the EBV-induced oncogenic process has been proposed in that its Gly-Ala repetitive sequence inhibited the specific cytotoxic T-cell recognition of immunodominant EBNA3B through a *cis*-acting mode (Levitskaya *et al.*, 1995). Several *in vitro* studies, however, demonstrated that LMP1 potentially confers tumorigenicity on non-lymphoid cells via transformation of established rodent fibroblasts (Wang *et al.*, 1985) and human keratinocytes (Fåhraeus *et al.*, 1990), inhibition of human epithelial cell differentiation (Dawson *et al.*, 1990), and induction of the functional epidermal growth factor receptor (Miller *et al.*, 1995). Such important findings strongly support crucial roles for LMP1 in the development of undifferentiated NPC, where the LMP1 positivity exceeds 80% of the cases at the trancriptional level (Brooks *et al.*, 1992; Chen *et al.*, 1995). The exact lack of LMP1 expression in EBV-positive gastric carcinomas, irrespective of their histological phenotypes, implies that LMP1 may not be necessary for the tumour, at least to sustain its already established malignant state. Rather, LMP1 might participate in an earlier stage of the tumour development and be down-regulated thereafter. Alternatively, the lack of LMP1 may reflect the result of

**Figure 3** RT–PCR analysis of promoter usage for EBNA gene transcription in EBV-positive gastric carcinomas. Results are shown by Et-Br staining (upper) and Southern hybridisation with $^{32}$P-labelled probes (lower). The structures of EBNA mRNA transcribed from the three different promoters Cp, Wp and Qp are shown by diagrams. Diagrammatic items and lanes represent the same ones as in the legend of Figure 1. The predicted sizes of each transcript are indicated at the left (bp).

**Figure 4** RT–PCR analysis of immediate-early BZLF1 gene expression in EBV-positive gastric carcinomas. Results are shown by Et-Br staining (upper) and Southern hybridisation with a $^{32}$P-labelled prope (lower). The splice structure of BZLF1 mRNA is shown by a diagram on the top of an Et-Br-stained gel. Diagrammatic items and lanes represent the same ones as in the legend of Figure 1. The predicted size of BZLF1 mRNA is indicated at the left (bp).

clonal selection of LMP1-negative tumour cells by immunological pressure because EBV-specific cytotoxic T cells are potentially directed against the viral latent proteins other than EBNA1 (Khanna *et al.*, 1992; Murray *et al.*, 1992). In fact, patients with EBV-positive gastric carcinoma have normally retained virus-specific immune T-cell responses (Imai *et al.*, 1994) as is the case with BL (Rooney *et al.*, 1985), in contrast to NPC patients (Moss *et al.*, 1983).

The EBV replication can be switched on in malignant or non-malignant epithelial proliferative lesions *in vivo*; the immediate-early BZLF1, the early and/or late antigens were detected in NPC (Cochet *et al.*, 1993), thymic carcinoma (Patton *et al.*, 1994) and AIDS-associated oral hairy leucoplakia (OHL) (Greenspan *et al.*, 1985). Similar viral cycle events were also observed *in vitro* by experimental EBV infection of cells of epithelial origin (Li *et al.*, 1992; Sixbey and Yao, 1992). There is a report of a patient with EBV-positive undifferentiated gastric carcinoma, in which a very small proportion of tumour cells expressed the BZLF1 protein, indicating that rare gastric carcinoma cells can potentially enter the lytic cycle (Niedobitek *et al.*, 1992). The present study in contrast, showed that neither the BZLF1 mRNA nor protein was found in gastric carcinoma tissues, results compatible with the absence of the early and viral capsid antigens as previously reported (Imai *et al.*, 1994). In addition, although EBV latency can be disrupted as epithelial cells differentiate, as shown by observations in OHL (Young *et al.*, 1991), none of the gastric carcinomas with various differentiations showed any evidence of virus production. LMP2A, detectably expressed in three of our seven cases, was shown to block calcium mobilisation in B cells, thereby suppressing EBV replication (Miller *et al.*, 1993, 1994). It is unknown whether the finding can be extended to epithelial cells; however, at least in certain EBV-positive gastric carcinomas, the absence of tumour cells entering the lytic cycle may be associated with LMP2A expression. It was inferred from our series of data that, unlike in NPC or OHL cells, virus replication was uncommon or abortive in gastric carcinoma, and that the vast majority of tumour cells carried EBV in a tightly latent fashion.

The strict localisation of EBV genomes and viral gene expression only in tumour cells and their monoclonality (Oda *et al.*, 1993; Imai *et al.*, 1994) strongly suggest that EBV may be indeed involved, not a passenger, in the development of virus-positive gastric carcinoma, although several questions remain to be resolved. Further studies are necessary to understand more direct oncogenic activities of EBV on normal gastric mucosal epithelia and a factor(s) enhancing them.

**Acknowledgements**
We thank Mr Hideki Nakamura for excellent technical assistance and Mr M Kim Barrymore for help with the manuscript. This work was supported in part by a scientific research grant from the Ministry of Education, Science, Sports and Culture, a grant-in-aid for the 10 year strategy for cancer control from the Ministry of Health and Welfare, Japan, and a research grant from the Suhara Memorial Foundation.

### References

BROOKS L, YAO QY, RICKINSON AB AND YOUNG LS. (1992). Epstein–Barr virus latent gene transcription in nasopharyngeal carcinoma cells: coexpression of EBNA1, LMP1, and LMP2 transcripts. *J. Virol.*, **66**, 2689–2697.

BROOKS LA, LEAR AL, YOUNG LS AND RICKINSON AB. (1993). Transcripts from the Epstein–Barr virus *Bam*HI A fragment are detectable in all three forms of virus latency. *J. Virol.*, **67**, 3182–3190.

BUSSON P, CCCOY R, SADLER R, GILLIGAN K, TURSZ T AND RAAB-TRAUB N. (1992). Consistent transcription of the Epstein–Barr virus LMP2 gene in nasopharyngeal carcinoma. *J. Virol.*, **66**, 3257–3262.

CHEN F, HU LF, ERNBERG I, KLEIN G AND WINBERG G. (1995). Coupled transcription of Epstein–Barr virus latent membrane protein (LMP)-1 and LMP-2B genes in nasopharyngeal carcinomas. *J. Gen. Virol.*, **76**, 131–138.

COCHET C, MARTEL RD, GRUNEWALD V, BOSQ J, COCHET G, SCHWAAB G, BERNAUDIN JF AND JOAB I. (1993). Expression of the Epstein–Barr virus immediate early gene, BZLF1, in nasopharyngeal carcinoma tumor cells. *Virology*, **197**, 358–365.

COUNTRYMAN J, JENSON H, SEIBL R, WOLF H AND MILLER G. (1987). Polymorphic proteins encoded within BZLF1 of defective and standard Epstein–Barr viruses disrupt latency. *J. Virol.*, **61**, 3672–3679.

DAWSON CW, RICKINSON AB AND YOUNG LS. (1990). Epstein–Barr virus latent membrane protein inhibits human epithelial cell differentiation. *Nature*, **344**, 777–780.

DEACON EM, PALLESEN G, NIEDOBITEK G, CROCKER J, BROOKS L, RICKINSON AB AND YOUNG LS. (1993). Epstein–Barr virus and Hodgkin's disease: transcriptional analysis of virus latency in the malignant cells. *J. Exp. Med.*, **177**, 339–349.

DIMERY IW, LEE JS, BLICK M, PEARSON G, SPITZER G AND HONG WK. (1988). Association of the Epstein–Barr virus with lymphoepithelioma of the thymus. *Cancer*, **61**, 2475–2480.

FÅHRAEUS R, RYMO L, RHIM JS AND KLEIN G. (1990). Morphological transformation of human keratinocytes expressing the LMP gene of Epstein–Barr virus. *Nature*, **345**, 447–449.

GILLIGAN K, SATO H, RAJADURAI P, BUSSON P, YOUNG L, RICKINSON A, TURSZ T AND RAAB-TRAUB N. (1990). Novel transcription from the Epstein–Barr virus terminal EcoRI fragment, DIJhet, in a nasopharyngeal carcinoma. *J. Virol.*, **64**, 4948–4956.

GREENSPAN JS, GREENSPAN D, LENNETTE ET, ABRAMS DI, CONANT MA, PETERSEN V AND FREESE UK. (1985). Replication of Epstein–Barr virus within the epithelial cells of oral 'hairy' leukoplakia, an AIDS-associated lesion. *N. Engl. J. Med.*, **313**, 1564–1571.

HARABUCHI Y, YAMANAKA N, KATAURA A, IMAI S, KINOSHITA T, MIZUNO F AND OSATO T. (1990). Epstein–Barr virus in nasal T-cell lymphomas in patients with lethal midline granuloma. *Lancet*, **335**, 128–130.

HERBST H, DALLENBACH F, HUMMEL M, NIEDOBITEK G, PILERI S, MULLER LN AND STEIN H. (1991). Epstein–Barr virus latent membrane protein expression in Hodgkin and Reed–Sternberg cells. *Proc. Natl Acad. Sci. USA*, **88**, 4766–4770.

IMAI S, KOIZUMI S, SUGIURA M, TOKUNAGA M, UEMURA Y, YAMAMOTO N, TANAKA S, SATO E AND OSATO T. (1994). Gastric carcinoma: monoclonal epithelial malignant cells expressing Epstein–Barr virus latent infection protein. *Proc. Natl Acad. Sci. USA*, **91**, 9131–9135.

KERR BM, LEAR AL, ROWE M, CROOM CD, YOUNG LS, ROOKES SM, GALLIMORE PH AND RICKINSON AB. (1992). Three transcriptionally distinct forms of Epstein–Barr virus latency in somatic cell hybrids: cell phenotype dependence of virus promoter usage. *Virology*, **187**, 189–201.

KHANNA R, BURROWS SR, KURILLA MG, JACOB CA, MISKO IS, SCULLEY TB, KIEFF E AND MOSS DJ. (1992). Localization of Epstein–Barr virus cytotoxic T cell epitopes using recombinant vaccinia: implications for vaccine development. *J. Exp. Med.*, **176**, 169–176.

KLEIN G, LINDAHL T, JONDAL M, LEIBOLD W, MENEZES J, NILSSON K AND SUNDSTRÖM C. (1974). Continuous lymphoid cell lines with characteristics of B cells (bone marrow-derived), lacking the Epstein–Barr virus genome and derived from three human lymphomas. *Proc. Natl Acad. Sci. USA*, **71**, 3283–3286.

KORBJUHN P, ANAGNOSTOPOULOS I, HUMMEL M, TIEMANN M, DALLENBACH F, PARWARESCH MR AND STEIN H. (1993). Frequent latent Epstein–Barr virus infection of neoplastic T cells and bystander B cells in human immunodeficiency virus-negative European peripheral pleomorphic T-cell lymphomas. *Blood*, **82**, 217–223.

LEAR AL, ROWE M, KURILLA MG, LEE S, HENDERSON S, KIEFF E AND RICKINSON AB. (1992). The Epstein-Barr virus (EBV) nuclear antigen 1 *Bam*HI F promoter is activated on entry of EBV-transformed B cells into the lytic cycle. *J. Virol.*, **66,** 7461-7468.

LEVITSKAYA J, CORAM M, LEVITSKY V, IMREH S, STEIGERWALD-MULLEN PM, KLEIN G, KURILLA MG AND MASUCCI MG. (1995). Inhibition of antigen processing by the internal repeat region of the Epstein-Barr virus nuclear antigen-1. *Nature*, **375,** 685-688.

LEYVRAZ S, HENLE W, CHAHINIAN AP, PERLMANN C, KLEIN G, GORDON RE, ROSENBLUM M AND HOLLAND JF. (1985). Association of Epstein-Barr virus with thymic carcinoma. *N. Engl. J. Med.*, **312,** 1296-1299.

LI QX, YOUNG LS, NIEDOBITEK G, DAWSON CW, BIRKENBACH M, WANG F AND RICKINSON AB. (1992). Epstein-Bar virus infection and replication in a human epithelial cell system. *Nature*, **356,** 347-350.

LIEBOWITZ D AND KIEFF E. (1993). Epstein-Barr virus. In *The Human Herpesviruses*. Roizman B, Whitley RJ and Lopez C. (eds) pp. 107-172. Raven Press: New York.

MILLER G AND LIPMAN M. (1973). Release of infectious Epstein-Barr virus by transformed marmoset leukocytes. *Proc. Natl Acad. Sci. USA*, **70,** 190-194.

MILLER CL, LONGNECKER R AND KIEFF E. (1993). Epstein-Barr virus latent membrane protein 2A blocks calcium mobilization in B lymphocytes. *J. Virol.*, **67,** 3087-3094.

MILLER CL, LEE JH, KIEFF E AND LONGNECKER R. (1994). An integral membrane protein (LMP2) blocks reactivation of Epstein-Barr virus from latency following surface immunoglobulin crosslinking. *Proc. Natl Acad. Sci. USA*, **91,** 772-776.

MILLER WE, EARP HS AND RAAB-TRAUB N. (1995). The Epstein-Barr virus latent membrane protein 1 induces expression of the epidermal growth factor receptor. *J. Virol.*, **69,** 4390-4398.

MOSS DJ, CHAN SH, BURROWS SR, CHEW TS, KANE RG, STAPLES JA AND KUNARATNAM N. (1983). Epstein-Barr virus specific T-cell response in nasopharyngeal carcinoma patients. *Int. J. Cancer*, **32,** 301-305.

MURRAY RJ, KURILLA MG, BROOKS JM, THOMAS WA, ROWE M, KIEFF E AND RICKINSON AB. (1992). Identification of target antigens for the human cytotoxic T cell response to Epstein-Barr virus (EBV): implications for the immune control of EBV-positive malignancies. *J. Exp. Med.*, **176,** 157-168.

NIEDOBITEK G, HERBST H, YOUNG LS, ROWE M, DIENEMANN D, GERMER C AND STEIN H. (1992). Epstein-Barr virus and carcinomas. Expression of the viral genome in an undifferentiated gastric carcinoma. *Diag. Mol. Pathol.*, **1,** 103-108.

NONKWELO C, SKINNER J, BELL A, RICKINSON A AND SAMPLE J. (1996). Transcription start sites downstream of the Epstein-Barr virus (EBV) Fp promoter in early-passage Burkitt's lymphoma cells define a fourth promoter for expression of the EBV EBNA-1 protein. *J. Virol.*, **70,** 623-627.

ODA K, TAMARU J, TAKENOUCHI T, MIKATA A, NUNOMURA M, SAITOH N, SARASHINA H AND NAKAJIMA N. (1993). Association of Epstein-Barr virus with gastric carcinoma with lymphoid stroma. *Am. J. Pathol.*, **143,** 1063-1071.

PALLESEN G, HAMILTON DS, ROWE M AND YOUNG LS. (1991). Expression of Epstein-Barr virus latent gene products in tumour cells of Hodgkin's disease. *Lancet*, **337,** 320-322.

PATTON DF, RIBEIRO RC, JENKINS JJ AND SIXBEY JW. (1994). Thymic carcinoma with a defective Epstein-Barr virus encoding the BZLF1 trans-activator. *J. Infect. Dis.*, **170,** 7-12.

QU L AND ROWE DT. (1992). Epstein-Barr virus latent gene expression in uncultured peripheral blood lymphocytes. *J. Virol.*, **66,** 3715-3724.

ROGERS RP, WOISETSCHLAEGER M AND SPECK SH. (1990). Alternative splicing dictates translational start in Epstein-Barr virus transcripts. *EMBO J.*, **9,** 2273-2277.

ROONEY CM, RICKINSON AB, MOSS DJ, LENOIR GM AND EPSTEIN MA. (1985). Cell-mediated immunosurveillance mechanisms and pathogenesis of Burkitt's lymphoma. *IARC Scient. Publ.*, **60,** 249-264.

ROWE M, ROWE DT, GREGORY CD, YOUNG LS, FARRELL PJ, RUPANI H AND RICKINSON AB. (1987). Differences in B cell growth phenotype reflect novel patterns of Epstein-Barr virus latent gene expression in Burkitt's lymphoma cells. *EMBO J.*, **6,** 2743-2751.

SAMPLE J, BROOKS L, SAMPLE C, YOUNG L, ROWE M, GREGORY C, RICKINSON A AND KIEFF E. (1991). Restricted Epstein-Barr virus protein expression in Burkitt lymphoma is due to a different Epstein-Barr nuclear antigen 1 transcriptional initiation site. *Proc. Natl Acad. Sci. USA*, **88,** 6343-6347.

SCHAEFER BC, WOISETSCHLAEGER M, STROMINGER JL AND SPECK SH. (1991). Exclusive expression of Epstein-Barr virus nuclear antigen 1 in Burkitt lymphoma arises from a third promoter, distinct from the promoters used in latently infected lymphocytes. *Proc. Natl Acad. Sci. USA*, **88,** 6550-6554.

SCHAEFER BC, STROMINGER JL AND SPECK SH. (1995*a*). Redefining the Epstein-Barr virus-encoded nuclear antigen EBNA-1 gene promoter and transcription initiation site in group I Burkitt lymphoma cell lines. *Proc. Natl Acad. Sci. USA*, **92,** 10565-10569.

SCHAEFER BC, STROMINGER JL AND SPECK SH. (1995*b*). The Epstein-Barr virus BamHI F promoter is an early lytic promoter: lack of correlation with EBNA 1 gene transcription in group 1 Burkitt's lymphoma cell lines. *J. Virol.*, **69,** 5039-5047.

SHIBATA D, TOKUNAGA M, UEMURA Y, SATO E, TANAKA S AND WEISS LM. (1991). Association of Epstein-Barr virus with undifferentiated gastric carcinomas with intense lymphoid infiltration. Lymphoepithelioma-like carcinoma. *Am. J. Pathol.*, **139,** 469-474.

SHIMIZU N, TANABE-TOCHIKURA A, KUROIWA Y AND TAKADA K. (1994). Isolation of Epstein-Barr virus (EBV)-negative cell clones from the EBV-positive Burkitt's lymphoma (BL) line Akata: malignant phenotypes of BL cells are dependent on EBV. *J. Virol.*, **68,** 6069-6073.

SIXBEY JW AND YAO QY. (1992). Immunoglobulin A-induced shift of Epstein-Barr virus tissue tropism. *Science*, **255,** 1578-1580.

SU IJ, HSIEH HC, LIN KH, UEN WC, KAO CL, CHEN CJ, CHENG AL, KADIN ME AND CHEN JY. (1991). Aggressive peripheral T-cell lymphomas containing Epstein-Barr viral DNA: a clinicopathologic and molecular analysis. *Blood*, **77,** 799-808.

TAKADA K AND ONO Y. (1989). Synchronous and sequential activation of latently infected Epstein-Barr virus genomes. *J. Virol.*, **63,** 445-449.

TIERNEY RJ, STEVEN N, YOUNG LS AND RICKINSON AB. (1994). Epstein-Barr virus latency in blood mononuclear cells: analysis of viral gene transcription during primary infection and in the carrier state. *J. Virol.*, **68,** 7374-7385.

WANG D, LIEBOWITZ D AND KIEFF E. (1985). An EBV membrane protein expressed in immortalized lymphocytes transforms established rodent cells. *Cell*, **43,** 831-840.

YOUNG L, ALFIERI C, HENNESSY K, EVANS H, O'HARA C, ANDERSON KC, RITZ J, SHAPIRO RS, RICKINSON A, KIEFF E AND COHEN JI. (1989). Expression of Epstein-Barr virus transformation-associated genes in tissues of patients with EBV lymphoproliferative disease. *N. Engl. J. Med.*, **321,** 1080-1085.

YOUNG LS, LAU R, ROWE M, NIEDOBITEK G, PACKHAM G, SHANAHAN F, ROWE DT, GREENSPAN D, GREENSPAN JS, RICKINSON AB AND FARREL PJ. (1991). Differentiation-associated expression of the Epstein-Barr virus BZLF1 transactivator protein in oral hairy leukoplakia. *J. Virol.*, **65,** 2868-2874.